# An analysis of framing mechanisms used in alcohol industry submissions to an Australian national parliamentary inquiry

**DOI:** 10.1093/heapro/daaf223

**Published:** 2025-12-23

**Authors:** Megan Cook, Dan Anderson-Luxford, Paula O’Brien, Deborah Gleeson

**Affiliations:** Centre for Alcohol Policy Research, La Trobe University, Melbourne, Victoria 3086, Australia; Centre for Alcohol Policy Research, La Trobe University, Melbourne, Victoria 3086, Australia; Melbourne Law School, The University of Melbourne, Parkville, Victoria 3010, Australia; School of Psychology and Public Health, La Trobe University, Melbourne, Victoria 3086, Australia

**Keywords:** submissions, alcohol, policy, framing mechanisms, commercial industries

## Abstract

The aim of this study is to analyse alcohol industry submissions to an Australian national parliamentary inquiry to understand the industry arguments and their implications for alcohol policy-making, and to test, for the first time, the applicability of Campbell and colleagues’ typology of framing mechanisms to alcohol industry submissions. We undertook a directed content analysis to code policy positions and arguments made by industry actors in the ten industry submissions, followed by thematic analysis to examine coded data for patterned responses according to the framing mechanisms. We identified four framing mechanisms: ‘equating’, ‘contesting’, ‘dichotomizing’, and ‘cropping’, which alcohol industry submitters used to highlight their corporate social responsibility efforts, industry leadership, self-regulation and community partnerships, while undermining effective evidence-based public health policy action. Industry submitters consistently used the inquiry as an opportunity to make arguments that supported maintenance of the regulatory ‘status quo’ and the continued inclusion of commercial actors as partners in policy decision-making. We identified heightened and direct attacks on public health evidence not previously seen within the Australian context. While examinations of frames remain important, stepping back and examining the framing mechanisms and actions employed can also offer insights about how to critique the discursive strategies—not just the specific arguments—being utilized by industry. From this critique, it is possible to (i) understand how some frames and arguments have gained acceptance, and others have not, and (ii) to respond to dominant frames and arguments by exposing the flaws in the discursive techniques that underpin them.

Contribution to Health Promotion

- This analysis of alcohol industry submissions to the ‘Inquiry into the health impacts of alcohol and other drugs in Australia’ identified heightened attacks on public health evidence not seen previously within the Australian context.
- Stepping back and examining the framing mechanisms and actions can offer insights about how to critique the discursive strategies being put forward by commercial industries.
- From this critique, it is possible to (i) understand how some frames and arguments have gained acceptance and (ii) to respond to the dominant frames and arguments by exposing the flaws in the discursive techniques by which they have been formed.

## Introduction

Commercial entities use a variety of strategies variously referred to as ‘political practices’ (Gilmore *et al*. 2023) or ‘corporate political activities’ (Ulucanlar *et al*. 2023) to influence, mitigate or avert public policies that might adversely affect their profits and commercial sustainability. Strategies by health-damaging industries including tobacco (Ulucanlar *et al*. 2016), ultra-processed food (Campbell *et al*. 2020), and alcohol (McCambridge *et al*. 2018) tend to follow a similar ‘playbook’ to exert influence and protect their interests (Lacy-Nichols *et al*. 2022). One of several political practices used by commercial actors to oppose or weaken effective public health regulation and promote industry-favoured alternatives is making submissions to government or parliamentary consultation processes (Martino *et al*. 2017, Miller *et al*. 2021). Consequently, corporate political activities represent a significant point of intersection between business and government, with important implications for policy outcomes. Tracking these commercial practices has become an integral part of national and global public health surveillance and efforts to protect public health (Ghebreyesus 2023). To contribute to this effort, we sought to analyse alcohol industry submissions to a recent national parliamentary inquiry in Australia to understand the arguments the industry is making and the implications for alcohol policy-making.

In terms of alcohol specifically, previous research has focussed on analysing the frames, strategies and arguments used by alcohol industry actors to influence policy at the national level (e.g. Stafford *et al*. 2020, 2021, 2022, Miller *et al*. 2023) and international level (e.g. O'Brien *et al*. 2023, World Health Organization 2024a, 2024b, Dünnbier *et al*. 2025). One strategy involves building relationships with decision-makers and framing the issues in ways that emphasize individual-level responses and downplay the effectiveness of population-level approaches to alcohol-related harm; emphasize the negative economic impacts of any regulation; and promote weak evidence and mimic scientific critique (mimicking scientific critique is when an attempt is made to critique scientific evidence, but the methods used to do so are not scientifically rigorous (Miller et al. 2023)) (McCambridge *et al*. 2018, Stafford *et al*. 2020, Miller *et al*. 2023, Cott *et al*. 2025). Such industry frames, strategies and arguments have been identified across various alcohol policy issues, including drinking guidelines (Wilkinson 2012 ), advertising regulations (Stafford *et al*. 2020, 2021, 2022), pregnancy warning labels (Avery *et al*. 2016) and taxation (Cullen *et al*. 2019). In all these policy debates, the industry has sought to reassert its importance and the legitimacy of its inclusion in policy development processes (O'Brien *et al*. 2023, World Health Organization 2024a, 2024b).

### Actions and mechanisms for creating frames

By repeatedly framing an issue in particular ways, an industry can create ‘path dependencies’ in the minds of policy-makers and the public about what the problem is and what constitutes credible courses of action to address the problem (Hawkins and Holden 2014). While identifying frames used by individual industries (and across industries) has been common, Campbell *et al*. (2020) propose an approach which moves beyond ‘identifying’ frames to analysing the ‘mechanisms’ and ‘actions’ by which frames are created within current debates. In their analysis of industry submissions to an Irish Government consultation for the proposed introduction of a sugar sweetened beverage tax in 2018, Campbell *et al*. (2020) uncovered nine underlying ‘framing actions’ (categorized into four groups of ‘framing mechanisms’) that had been used by industry submitters to generate frames (detailed in the methods below).

The analysis of framing mechanisms and actions, rather than honing in and only considering the specific frames and arguments used by a particular industry on a particular topic, is said to ‘give insight across issues’ into ‘the organizational mechanics’ of creating a discourse around a subject matter, whether the matter be sugar or alcohol or fossil fuels (Campbell *et al*. 2020). Campbell *et al*. (2020) argue that understanding the ‘mechanics’ of framing may help to ‘deconstruct any given frame that becomes dominant in corporate discourse’. From this analytical perspective, the techniques, devices and logics of argumentation used by the industry to build the acceptance and salience of certain frames and positions are made ‘visible’. This visibility then opens up the industry's claims and arguments to systematic critique that, in turn, enables a comprehensive response or rebuttal, including by exposing the flaws or weaknesses in the discursive techniques by which these claims and arguments have been formed.

This framing mechanisms and actions approach was originally developed and applied within the context of food policy and revealed, for example, how often sugar industry actors using a ‘dichotomizing’ technique where people, their perspectives and options for change are divided into hard categories of ‘good’ and ‘bad’ (Campbell *et al*. 2020). Given the similar ‘playbook’ identified across different commercial sectors (Lacy-Nichols *et al*. 2022), the framing mechanisms and actions identified by Campbell *et al*. (2020) may also be relevant for examining the policy-related frames and arguments used by alcohol industry actors. In this article, we test, for the first time, the applicability of the Campbell and colleagues’ typology of framing mechanisms and actions to alcohol and we analyse the utility of this typology for research into corporate practices in relation to alcohol.

### Inquiry into the health impacts of alcohol and other drugs in Australia

In August 2024, the Commonwealth Parliament’s House of Representatives Standing Committee on Health, Aged Care and Sport commenced an inquiry into the health impacts of alcohol and other drugs in Australia. The Committee, chaired by an Australian Labor Party Member of Parliament, Dr Mike Freelander, aimed to review policy, treatment services, community programmes and the alcohol and other drug workforce to determine ‘whether the current settings appropriately support the prevention, reduction and recovery of alcohol and other drugs-related health harms on individuals, families and communities’ (Parliament of Australia 2024a). The specific terms of reference for the national inquiry were as follows (Parliament of Australia 2024b):

1. ‘Assess whether current services across the alcohol and other drugs sector is [sic] delivering equity for all Australians, value for money, and the best outcomes for individuals, their families, and society;
2. Examine the effectiveness of current programmes and initiatives across all jurisdictions to improve prevention and reduction of alcohol and other drug-related health, social and economic harms, including in relation to identified priority populations and ensuring equity of access for all Australians to relevant treatment and prevention services;
3. Examine how sectors beyond health, including for example education, employment, justice, social services and housing can contribute to prevention, early intervention, recovery and reduction of alcohol and other drug-related harms in Australia; and
4. Draw on domestic and international policy experiences and best practice, where appropriate’.

The Committee originally requested submissions to be made by September 2024, although submissions were accepted beyond this date, and no restrictions were placed on who could submit. The Committee published an issues paper but not a final report, as the inquiry lapsed when the Commonwealth Parliament was dissolved on 27 March 2025 before the federal election (Parliament of Australia 2025). Given the potential importance of this inquiry for shaping the future of alcohol policy (including prevention and treatment options) in Australia, we critically examine the framing mechanisms and actions used in submissions by the alcohol industry to this national inquiry. The main aim of this project is to understand which alcohol industry actors are engaging in this national inquiry and the framing mechanisms and actions used by industry actors in their written submissions. Based on this understanding, we then consider the implications of the political strategies of commercial actors for government policy-making in relation to alcohol in Australia.

## Materials and methods

Publicly available submissions were downloaded in late 2024 to early 2025 from the Inquiry webpage (*N* = 204). Submissions were received from a range of organizations and individuals, with most submissions received from health-focussed, non-governmental organizations. Four submissions were confidential and not able to be accessed. Table 1 shows the characteristics of all submissions received, excluding the four confidential submissions (*n* = 200).

**Table 1:** Characteristics of submissions.

	*N*	%
**Organization type**		
Non-governmental organization—health focussed	91	45.5
Individual—personal experience	30	15
Non-governmental organization—other	30	15
Academic organization or individual	23	11.5
Private sector—alcohol industry	10	5
Government	10	5
Other	6	3
**Focus of organization making the submission**		
Health	90	45
Alcohol and other drugs	66	33
Alcohol	33	16.5
Other drugs	11	5.5
**Submission page length**		
0.1–2 pages	10	5
3–4 pages	24	12
5–6 pages	27	13.5
7–9 pages	37	18.5
10+ pages	102	51

All submissions made by an ‘alcohol industry actor’ were included in the analysis (*n* = 10; see Table 2). We defined an ‘alcohol industry actor’ as:

1. a manufacturer, distributor, or retailer of alcohol products or
2. an organization formed to represent the interests of two or more alcohol manufacturers, distributors or retailers (such a peak trade body for spirits manufacturers) or
3. an organization formed to undertake ‘corporate social responsibility’ initiatives in the interests of the ‘alcohol industry actors’ referred to in (1) and (2) above, and funded wholly and primarily by one or more such actors [also referred to as ‘Social Aspects/Public Relations Organizations (Babor 2009)], or
4. an organization formed to operate an industry-wide self-regulatory scheme for one or more of the ‘alcohol industry actors’ referred to (1) and (2) above, or
5. an individual or organization expressly commissioned or retained by one or more of the ‘alcohol industry actors’ referred to above in (1)–(4) to make a submission to the Inquiry.

**Table 2:** Industry submissions.

Industry organization	Description of organization	Scope (National or state organization)	Page length
Alcohol Beverages Australia	Peak body^b^ representing alcohol industry manufacturers, distributers, and retailers	National	17 pages
Brewers Association Australia	Peak body representing the beer industry	National	9 pages
Clubs Australia	Coalition of state and territory associations representing licenced clubs across Australia and New Zealand	National	2 pages
Independent Brewers Association	Peak body representing independently-owned brewers and their supply chain partners	National	4 pages
Retail Drinks Australia	Peak body representing Australia’s packaged retail liquor stores	National	16 pages
Spirits & Cocktails Australia	Peak body for major spirits manufacturers	National	32 pages
ABAC Scheme Limited	Alcohol industry self-regulatory scheme for alcohol marketing	National	5 pages
DrinkWise	Alcohol industry education and information organization	National	16 pages
Harvest Advisory & Research^a^	Independent consultancy company	N/A	18 pages
Australian Hotels Association (WA)	Peak membership association for the hotel and hospitality industry in Western Australia	State	14 pages

^a^Author of a report commissioned by Alcohol Beverages Australia. ^b^Peak bodies are organizations that represent a specific industry, profession, or community and their interests.

We did not search for submissions by individuals or organizations that are funded by an alcohol industry actor but whose purposes and activities are not primarily in the service of the alcohol industry. Alcohol industry submissions were identified based on readily identifiable information (e.g. name or organization information provided in the submission). Six of the 10 alcohol industry submissions were ten or more pages in length, although given the variation in formatting and use of tables and figures, this information only provides a broad sense of the length of submissions.

### Data analysis

We undertook a directed content analysis (Hsieh and Shannon 2005) and a thematic analysis (Braun and Clarke 2006), both of which entailed a process of deductive coding. We applied a previously developed and validated coding schedule (Dwyer *et al*. 2022) to code the policy positions and arguments made by industry actors. Dwyer and colleagues’ (2022) coding schedule was developed to comprehensively analyse policy submissions in the context of alcohol and other drugs, and builds on previous research (McCambridge *et al*. 2013, Stafford *et al*. 2020). This schedule was developed in the context of a study of alcohol industry submissions made to a World Health Organization (WHO) consultation, and included several codes focussed on the WHO SAFER initiatives which were not relevant to this study and were not included for this analysis. Additionally, in the current study, the primary source for any evidence referred to in the industry submissions was also examined to determine the accuracy of the submitter’s reporting and the source of the evidence (e.g. industry-funded bodies). As coding progressed, we added one new code to the schedule to capture mentions of priority populations. Full submissions from alcohol industry actors were independently double coded (M.C., D.A.-L.) in NVivo. The team met to discuss coding as it progressed, resolving any discrepancies through discussion.

A further stage of thematic analysis then examined the coded data for patterned responses according to Campbell and colleagues’ (2020) nine framing actions. These were categorized into the four framing mechanisms as seen in Table 3. The data for this study is publicly available submissions, and no ethics approval was required.

**Table 3:** Framing mechanisms and actions.

Framing mechanism	Definition	Framing action	Definition
1. Equating	Involves seeking parity between incommensurable frames, i.e. claiming that industry stakeholders are legitimate contributors that should be on an equal footing to other stakeholders, and conflating different frames in strategically useful ways.	1.a Demanding parity	Involves the assertion by commercial actors that they be considered on the same level as any other stakeholders in decision-making processes.
		1.b Conflating	Involves the combination of elements from several different frames, thereby creating some confusion about corporate activities. A clear example of this is conflating the global brand and the global corporate structure which ‘makes it difficult to discern the global infrastructure and agency of these industry actors’. (Campbell *et al*. 2020, p. 5).
2. Contesting	Involves processes which can undermine the structure of a frame so that its boundaries become ‘permeable’ and new logics can enter. It can involve ‘co-opting’ arguments that originate from public health or ‘exiting’ from past frames that the industry no longer wants to use, particularly if they are no longer palatable in some way.	2.a Co-opting	‘The process where a system or social movement assimilates its opposite’ (Campbell *et al*. 2020, p. 4).
		2.b Exiting	Refers to a commercial actor’s disassociation with a previously used frame.
3. Dichotomizing	Involves breaking a frame apart into distinct factions. This often is done by ascribing positive characteristics to industry while indirectly (by comparison) suggesting negative characteristics about public health actors and government.	3.a Attributing	Involves ‘processes where actors tie objects to effects in order to simplify their environments, and understand, predict and control the behaviour of other actors’ (Campbell *et al*. 2020, p. 3).
		3.b Boomeranging	Involves turning an opponent’s own logic against them, either by taking their premise literally or by revealing how it fails to support their own arguments. Using the example of causality, Campbell and colleagues (2020, p. 4) describe how ‘Through boomeranging, industry uses public health's own research quality criteria for causality against it while deflecting attention from the major shortcomings of research which industry itself relies on for its counter-argument’.
4. Cropping	Involves removing part of the frame to draw attention to particular focus areas (rather than the whole picture) and shift blame to others outside the frame.	4.a Jockeying	Involves attempting to make a special or unique case for their brand, service, or industry. This can result in several distinct cases being put forward.
		4.b Siloing	Involves the ‘funnelling of logics’ so that the actor can inhabit two opposing positions simultaneously, for example problem solver and problem maker or mighty but also modest.
		4.c Blame-shifting	Involves the ‘singling out of another actor or object to carry the burden of negativity within a frame, so that attention is focused on the most blame-worthy and the industry actor may reap the ‘lesser of two evils effect’.

## Results

Here we present findings focussed on the mechanisms used in alcohol industry submissions to the national inquiry. These four mechanisms are: ‘equating’, ‘contesting’, ‘dichotomizing’, and ‘cropping’ (Campbell *et al*. 2020). We also consider the specific actions used to operationalize the mechanisms.

### Equating: demanding parity (1.a)

We identified a consistent assertion in the submissions that commercial actors should be included in decision-making and governance as equal stakeholders, alongside public health organizations and government—an argument that was made by emphasizing the positive contribution the industry makes to Australian society. At the local community level, industry submissions highlighted their support of local running clubs, book clubs and ‘sip and sweat’ Pilates classes (Independent Brewers Association). These wellbeing initiatives were lauded as building social connection to combat loneliness and isolation and addressing social issues around mental health.

Industry submissions also described their progress developing and implementing ‘world class’ voluntary codes for responsible alcohol advertising (e.g. ABAC Scheme Limited), Retail Drinks Guidelines for Responsible Product Ranging Decisions and a code of conduct for responsible online sale and delivery (Retail Drinks Online Code). The development of this Online Code was described as having ‘been internationally recognized as best practice’ (Alcohol Beverages Australia). Such initiatives were used by industry to demonstrate its leadership and the success of its self-regulation. Additionally, seven of the ten industry submissions highlighted the socio-economic importance of the alcohol industry and its contributions to Australia in terms of employment, e.g.:

Our industry contributes $15.5 billion in added value to the Australian economy, supporting 5700 spirits manufacturing jobs and a further 45,400 jobs in spirits wholesale, retail and hospitality. An additional 48,700 indirect jobs are supported throughout our supply chain. (Spirits & Cocktails Australia)

Relatedly, several submissions also referred to the alcohol industry’s significant (indirect) economic contribution to treatment and healthcare related to alcohol and other drugs, e.g. ‘Australian spirits excise delivers a signiﬁcant ﬁnancial contribution to the national health system.’ (Spirits & Cocktails Australia).

As part of the case for parity of treatment of alcohol industry actors in the policy process, industry actors described themselves as equal stakeholders who are proactively ‘contributing to a positive drinking culture’ (Alcohol Beverages Australia), with submissions emphasizing that the industry has taken steps to promote ‘responsible consumption’ and reduce harm. Support was given in four submissions for the work of DrinkWise, including its education campaigns that aim to create awareness of not drinking during pregnancy, of parental influence on young people’s drinking, and the importance of moderate consumption.

After highlighting and establishing the positive contributions of the alcohol industry, submissions then further demanded parity with government and public health actors by focussing on opportunities for collaboration, partnership and industry involvement in governance. For example, Alcohol Beverages Australia discussed supporting local projects that are opportunities to promote responsible drinking and reiterate a commitment to work alongside governments and communities: ‘The alcohol industry is committed to working with governments, service providers and communities to reduce harmful drinking in Indigenous communities’. Finally, the National Alcohol Strategy 2019–28 was also highlighted as an example of successful collaboration that included industry, e.g.:

The National Alcohol Strategy 2019–2028 has been successful in bringing together the Commonwealth, State and Territories, the alcohol industry and other stakeholders to tackle harmful alcohol consumption and reduce underage consumption. (Brewers Association Australia)

### Equating: conflating (1.b)

We also identified another type of ‘equating’ work—described by Campbell *et al*. (2020) as ‘conflating’. While all commercial actors who participated in this inquiry were national or state-based organizations, we saw a focus in several submissions on localized engagement, for example, ‘As member-owned, not-for-profit organizations, clubs are deeply embedded in their communities, committed to fostering safe and responsible drinking cultures, and actively support their communities through a range of social, charitable, and volunteer initiatives that contribute to local wellbeing’. (Clubs Australia). Similarly, DrinkWise discussed its work with Indigenous Australian communities. In doing so, industry submitters seem to be representing themselves as embedded in local communities making it difficult for the public to discern the corporate power and agency of the industry actors.

### Contesting: co-opting (2.a)

Next, we identified how commercial actors co-opt symbolically important arguments and language from public health in order to derive opposing conclusions, as part of contesting the policy terrain. As part of this action, industry submitters used language that was both ‘vague’, but ‘emotionally powerful’ (Campbell *et al*. 2020), and that worked to position industry actors as aligned with broader social values held by the community and as leaders willing to take action to support communities and individuals. For example, DrinkWise discussed how their overall approach was underpinned by a ‘whole-of-community’ approach whilst arguing only for targeted solutions, aimed at priority populations, and involving a range of actors, including the industry. Their language around ‘systems level’ and ‘whole of community approaches to change’ seems to draw on the WHO Global Alcohol Action Plan (World Health Organization 2020, p. 15).

Other vague but powerful use of terminology identified in six industry submissions included poorly defined terms such as ‘moderate’ or ‘moderation’ to imply a level of acceptable consumption that any responsible drinker would observe. As Campbell and colleagues (2020) attest, the ‘performative value’ of language such as ‘moderate’ is a crucial part of co-option. Five of these industry submissions emphasized the benefits of moderate consumption, e.g.:

It is also important to recognise the social and psychological beneﬁts of alcohol. From a social perspective, alcohol can help reinforce social bonds, enhances community engagement and often plays a role in celebrating signiﬁcant life events such as births, deaths, graduations, and marriages, as well as social functions. (Spirits & Cocktails Australia)

Additionally, in setting out the ‘problem’, Spirits and Cocktails Australia stated that, ‘More than three quarters of Australians consume alcohol as part of a balanced lifestyle’. While clear definitions of moderate consumption were absent, as rhetorical devices, the language of ‘moderation’, ‘balanced’ and ‘choice’ (as seen, e.g. in the DrinkWise submission) reinforces the responsibility of individuals to reduce any experience of harm. While these terms are used by public health when narrowly defined and purely descriptive (i.e. not accompanying implications around responsibility), industry co-option allows the industry to ‘share’ concerns with public health and reinforce individual responsibility.

Other language identified across submissions that attempted to position industry entities as trustworthy, socially conscious actors included self-referential terms such as ‘sensible’, and ‘responsible’. For example, in its vision statement on the first page of its submission, Brewers Association of Australia claimed to be ‘a leading voice for sensible, responsible and workable policy solutions for our sector and the community’. The adjectives used here position the industry as concerned actors whose values align with the community and which contests the role of public health as responsible and socially conscious (Campbell *et al*. 2020).

### Contesting: exiting (2.b)

While in previous analyses of alcohol industry submissions, the J-Shaped curve was a key argument drawn on by the industry around the benefits of alcohol consumption (WHO 2024a), we identified some distancing from this line of reasoning in the submissions to the national inquiry. In our analysis, only one industry submitter, Spirits and Cocktails Australia, included outdated and weak evidence around the J-Shaped curve to claim that moderate alcohol consumption can have health benefits.

### Dichotomizing: attributing (3.a)

In the ten submissions analysed, we did not identify any evidence of actors tying objects to effects in the manner described by Campbell *et al*. (2020), however submissions heavily utilized the other dichotomizing action, boomeranging.

### Dichotomizing: boomeranging (3.b)

Submitters used this action by adopting academic models of critique and demanding demonstrations of causality not possible in the type of correlation studies or reports underpinning the evidence base. This boomeranging was most evident in the Harvest Advisory and Research submission ‘Considering Four Reports Making Claims about Alcohol: A Concise and Objective Opinion’ commissioned by Alcohol Beverages Australia. This industry-funded submission aimed to dissect four reports by two public health organizations (the Foundation of Alcohol Research and Education and Alcohol Change Australia) and focussed almost exclusively on critiquing the methodological rigour and bias in the reports, without situating the critique within the broader public health literature. The Harvest submission relies on valid frameworks of scientific critique, such as the Critical Appraisal Checklist and the Total Survey Error (TSE) framework, to draw attention to legitimate issues with sampling (i.e. non-probability panel samples limit generalizability), lack of transparency (e.g. absence of details concerning ethics, reimbursement, statistical methodology, etc.) and overgeneralized conclusions (e.g. assuming population-level implications when generalizability is limited) in the four reports. However, the conclusions drawn by the Harvest submission from these criticisms of the four reports are not in line with scientific standards and they warrant assessment. For example, the Harvest submission concludes that:

It is HAR’s opinion that these limitations are so substantial that the alleged findings and claims made across the reports should be publicly withdrawn and apologies issued. These reports are unsuitable for policymaking or public health and are likely to mislead various media and publics. (Harvest Advisory and Research)

The Harvest submission offers an unequivocal dismissal of the four reports, with no acknowledgement of their potential contributions (as is customary with pilot studies, e.g.), characterizing the studies as entirely without merit, ‘unsuitable for policy-making’, and ‘junk science’ (Harvest submission). In contrast, scientific standards (e.g. including the TSE framework) emphasize caution when interpreting findings from non-probability samples but do not advocate for outright dismissal based on the outlined methodological shortcomings. Similarly, where omissions occur, scholarly convention dictates providing the opportunity for clarification, reflecting the shared scientific aim of testing and strengthening the robustness of the evidence base. Thus, the framing and conclusions presented in the Harvest submission appear to reflect a partial and selective interpretation of the reports under consideration.

Moreover, this selective critique reflects a broader strategy of demanding methodological perfection when the evidence is contrary to the industry’s agenda, while simultaneously relying on weak evidence when it supports the industry’s key arguments. For example, Alcohol Beverages Australia cite a study, partly funded by industry, which concluded that ‘the social consumption of alcohol may thus have the same effect as many other social activities, such as laughter, singing and dancing’ (Dunbar *et al*. 2017). This conclusion is largely erroneous when assessed against the scientific standards demanded by the industry in other contexts: (i) it is based on a non-probability online panel, (ii) response rates and recruitment details are not adequately reported, and (iii) causal associations are implied from cross-sectional observational data. The specific claim of equivalence between social alcohol consumption and other endorphin-activating activities is predicated on a speculative and untested mechanism (i.e. that alcohol causally produces social bonding through endorphin release), rather than being supported by direct empirical evidence.

Simultaneously, other industry submissions referenced a small number of self-published research or consultancy reports often with similar methodological biases and limitations as critiqued by the Harvest submission. For example, Retail Drinks Australia cited findings from an unpublished report by Data Analysis Australia (DAA) to argue that alcohol policy should not be developed on ‘the flawed assumption that there is an intrinsic link between [alcohol retail outlet] density and harm’. Despite being unpublished, and therefore immune from independent scrutiny, the submission claimed that ‘the DAA research methodology provides distinct improvement on previous studies on liquor outlet density’ and reported that ‘socio-economic factors were up to 200 times more significant as a contributor to levels of harm than outlet density’. It remains unclear whether the DAA report was commissioned directly by the industry, but Retail Drinks Australia had access to the findings which it stated were ‘currently under peer review’.

### Cropping: jockeying (4.a)

Jockeying, along with the other cropping action (siloing) were less commonly noted in the submissions than the other framing actions included in the framework developed by Campbell *et al*. (2020). But one example of jockeying was seen in the emphasis in submissions on the positive contributions of the alcohol industry, including for employment and the economy, in which submitters were attempting to make a unique case for their service, beverage or industry. For example, the Independent Brewers Association states on its opening page that ‘Independent breweries are overwhelmingly small to medium businesses that employ locals and give back to their communities’, contrasting with other industry submitters who make a case for the significance of their business and employment portfolio (see examples highlighting the socio-economic importance of the industry above).

### Cropping: siloing (4.b)

As described above, industry submitters stated that they were committed to working alongside government and communities to reduce alcohol-related harm, commonly supporting the work of DrinkWise to do so. Successes highlighted in the DrinkWise submission of their work included the claim, ‘33% of parents who had intended to supply alcohol to their underage teenagers decided they would not after seeing the [DrinkWise] campaign’. However, this siloing action ignores the role of the alcohol industry in producing and selling alcohol. As such the industry could be seen as both the cause of, and solution to, the problem.

### Cropping: blame-shifting (4.c)

In our analysis, we saw cropping in the form of blame-shifting chiefly in discussions of illicit alcohol, which was raised in four submissions as an issue for government, business and the public’s health. For example, ‘Estimates indicate that illicit alcohol accounts for approximately 7.5% of the total per capita alcohol consumption in Australia, with severe implications for tax, public health and safety and the business viability of legitimate retailers and producers’ (Retail Drinks Australia). The submission crops out the complexity of the topic of ‘illicit’ alcohol, with no definition provided for the term ‘illicit alcohol’ (Australian Taxation Office 2025) and no distinction drawn between ‘illicit’ and ‘unrecorded’ alcohol (WHO 2018). There were also concerns that the unregulated production of illicit alcohol may cause health harms, e.g.

Not only is the Commonwealth therefore losing considerable revenue, but the illegal alcohol produced and sold is dangerous. The illegal operations do not comply with Australia’s rigorous regulatory regime regarding product safety. (Alcohol Beverages Australia)

In another example, blame was shifted to other industries. For example, ‘At a time when society is increasingly disconnected, our taprooms and brewpubs serve as the place where people can come together over a meal and a hand-crafted beer to discuss ideas, converse about society and feel connected—**without being surrounded by gambling**’. (bold in original text; Independent Brewers Association). Here, the gambling industry, rather than alcohol, was portrayed as posing a threat to the social corporate good.

## Discussion

In our analysis of alcohol industry submissions to the ‘Inquiry into the health impacts of alcohol and other drugs in Australia’, we found the consistent use of arguments that have been identified in previous analyses of alcohol industry lobbying and that can serve to undermine effective, evidence-based efforts to reduce the health and other impacts of alcohol and other drugs (e.g. McCambridge *et al*. 2018, Dwyer *et al*. 2022, Miller *et al*. 2023). Unlike previous inquiry processes where many industry actors participated (e.g. Miller *et al*. 2023), industry actors engaging in this inquiry were few in number and were mainly national peak bodies (i.e. membership organizations that represent a specific industry segment of the alcohol market). This may be due to the focus of the inquiry on health impacts and health services (Parliament of Australia 2024b), rather than being about a particular proposed policy intervention of concern to the alcohol industry. It may also reflect a decision by the industry to participate in these types of inquiries through their peak bodies and speak with ‘one voice’, rather than having individual producers and retailers make submissions. It may also be that the alcohol industry views other (more private) avenues of communicating with members of parliament as more beneficial than participation in public inquiries (Lacy-Nichols *et al* 2023, Belot 2025).

Despite the small number of industry submissions, the alcohol industry submitters consistently used this inquiry as an opportunity to make arguments that supported the regulatory ‘status quo’ being maintained with respect to alcohol, and similar to previous studies (e.g. O'Brien *et al*. 2023), the alcohol industry argued against its marginalization from decision-making by emphasizing the positive contribution the industry makes to Australian society. It takes government considerable effort to develop, pass and implement new legislation, and governments at the federal, state and territory levels are facing a range of pressing social and economic problems that compete for their limited capacity and attention. It is easier for governments not to change regulatory settings than to embark on complicated and contested regulatory reform processes. The alcohol industry in Australia appears focussed on convincing governments that they do not need to expend regulatory effort on alcohol—and this position has been largely effective in the past in Australia where there has been very little alcohol policy development over the past several decades (with the critical exception being alcohol and pregnancy warning labels (O'Brien 2021)).

Industry sought to ‘equate’ themselves with public health interests and institutions by highlighting their successful corporate social responsibility efforts, industry leadership, self-regulation and community partnerships. Importantly, through this framing mechanism of equating, industry sought to make the case for commercial actors to be included as partners in policy decision-making. Preceding calls for the industry’s inclusion with countless examples (across all levels of society) of the industry ‘doing good’, deflects attention from the significant harm it causes and makes its exclusion from policy-making seem illogical.

Calls for ‘whole of community’ responses also echo the WHO Global Alcohol Action Plan and speak to a framing action of ‘co-opting’ language that is used by public health organizations. The industry seemed to use the term to again legitimize their place in the society and in policy-making processes: if the ‘whole’ community needs to be involved in solving the problem of alcohol, then of course that includes the industry. But this is not what is meant by the terms ‘whole of community’ or ‘whole of society’ from a public health perspective. These terms refer to an approach involving cooperation across levels and institutions of government and with civil society and communities—to the ‘exclusion’ of industry actors—to address alcohol consumption and related harm (WHO 2018, Campbell *et al*. 2020). These terms also describe an approach characterized by a focus on system-level interventions which seek to change the underlying structures that contribute to the problem, not just individual behaviours (e.g. Terry and Burris 2023). In such a public health approach, the industry’s conduct is regarded as part of the system that contributes to the harm, but the solution is not to allow the industry to regulate itself. Rather, the industry’s conduct is to be regulated by government to remove or reduce the impact that the industry’s behaviour has on the public’s health (WHO 2024b).

We saw the industry commonly promote targeted measures aimed at priority populations and illegal alcohol over population-wide policies, often without sufficient evidence to support these approaches. The terms ‘balanced’ and ‘lifestyle’ perpetuate a disproportionate focus on the individual and distract from the role of social and commercial determinants that shape an individual’s life and circumstances. Previous research has identified this as a common strategy (Miller *et al*. 2021, 2023), and shown that the industry’s self-regulatory efforts, in particular with alcohol labelling and marketing, have been ineffective in Australia and have arguably stalled effective policy-making by government (O'Brien 2021, 2023).

‘Dichotomizing’ actors as either positive or negative allows industry actors to avoid directly attacking public health research (Campbell *et al*. 2020), but it is a framing mechanism that works to undermine public health arguments regardless. The action of ascribing industry actors and goals with positive characteristics, by default, casts the other actor (in this case, any public health actors whether researchers, advocates or government agencies) as the ‘bad guy’ for holding back partnership and further benevolence on the part of the industry. Attempts to legitimize the role of industry in policy submissions have been identified in previous research (O'Brien *et al*. 2023, World Health Organization 2024a, 2024b), and are notably important because positioning the industry as part of the solution can enhance its credibility, create an imperative for government to include industry in reform processes and thereby reduce the likelihood of effective regulation (Clare *et al*. 2022). Being part of the solution, rather than the problem, has been a key long term alcohol industry strategy (Madden *et al*. 2023)—it involves defining the problem as nothing to do with alcohol *per se* or the conduct of the industry but rather to do with problematic individual drinking behaviour. This is also an example of framing by ‘cropping’ and ‘blame-shifting’ (Campbell *et al*. 2020) and aligns with the findings of previous research (Mialon and McCambridge 2018) which has found an industry focus on individual responsibility for drinking-related harms to self and others, and on education for personal behaviour change as the solution through programmes designed and delivered by industry-funded public relations organizations, like DrinkWise.

Our findings also align with a significant body of Australian and international research showing how the industry attempts to weaponize opponents’ logic through adopting elements of academic critique (McCambridge *et al*. 2018, Petticrew *et al*. 2018, Stafford *et al*. 2020, Miller *et al*. 2023, Cott *et al*. 2025). As others have documented, this type of ‘boomeranging’ action by industry is used to undermine public health evidence and to cast public health solutions as weak and ineffective. However, we suggest that the direct attacks on legitimate science identified in submissions to the National Inquiry (e.g. the Harvest submission) represent a new alcohol industry strategy that has not been seen previously within the Australian context. It appears that the primary purpose of the Harvest submission is to denigrate the relevance of the findings from the reports by the Foundation for Alcohol Research and Education and Alcohol Change Australia to policy discussions, including the National Inquiry itself. Commercial actors are agile and dynamic, constantly adapting strategies and evolving in response to shifting market and political pressures. While other framing mechanisms and actions identified in our study have been seen previously in political activities by the alcohol industry, this critique of public health evidence may suggest a new and more hostile direction that the Australian alcohol industry is taking in response to pressures for better regulation of alcohol (see examples of suppression of public health evidence from analyses of the tobacco industry such as that by Ulucanlar *et al*. 2016).

Overall, the framing actions of attributing, jockeying, and blame-shifting were least common in this analysis, while dichotomizing, boomeranging, co-opting and exiting were identified as most common (see Supplementary File S1). While Campbell and colleagues identified industry actors ‘exiting’ or disassociating with narratives of personal responsibility in their study of policy processes relating to sugar, we saw a sustained focus on individual (or personal) responsibility. In our sample, exiting was primarily identified through industry actors moving away from relying on evidence around the J-Shaped curve, with only one industry submission drawing on this evidence. It has been suggested by public health advocates that by continuing to rely on outdated evidence around the J-Shaped curve, the industry is seeking to create confusion, particularly among the public about the health risks from alcohol (WHO 2024a, 2024b, Dünnbier *et al*. 2025). While blame-shifting was not common in this analysis, it importantly works alongside the other actions to shift the focus from the industry, and to create a separate blameworthy actor—the irresponsible individual drinker—to whom the problems from alcohol could be attributed (in this case, illicit alcohol).

While examinations of frames and arguments remain important, stepping back and examining (as we have successfully done here) the framing mechanisms and actions that underpin these frames and arguments can also offer insights for researchers, policy-makers, and advocacy organizations about how to critique the discursive strategies—and not just the specific claims—being put forward by industry. We believe this is important in instances such as these where framing mechanisms or actions that suggest parity or equivalence (equating, boomeranging, co-opting) between public health and commercial actors and arguments can stifle policy development that serves the public’s interests. If policy-makers treat everyone before them as equal in terms of expertise and legitimacy, then there is the potential for policy paralysis or policy misdirection.

More broadly, in line with the findings from Campbell *et al*. (2020) on the food industry, this study demonstrates the various ways in which the alcohol industry deploys framing mechanisms to ‘assert control of the narrative’ and denigrate public-health orientated arguments for alcohol policy reform. For this reason, it is important to consider potential avenues for the implementation of safeguards that ensure decision-making processes are not unduly biased by arguments which run contrary to evidence-based public policy. Moreover, further deconstruction of the framing mechanisms and actions by industry actors can help improve literacy and awareness among government and policy makers, thereby facilitating balanced and critical appraisals of submissions to future inquiries and consultation processes.

### Limitations & future research

There are several limitations which need to be kept in mind when interpreting these results. Firstly, our sample included a number of organizations within the definition of an ‘alcohol industry actor’, but we did not examine differences in submissions by actor sub-type. Secondly, while DrinkWise describes itself as an independent ‘evidence-based social change organization dedicated to creating a safer and healthier drinking culture in Australia’, the fact that its work ‘is funded primarily through voluntary alcohol industry contributions’ with 18 alcohol manufacturers, distributors or retailers listed as funders, led us to include it as an ‘alcohol industry actor’ (DrinkWise 2024). We note that DrinkWise and similar organizations in other countries have been categorized as part of the alcohol industry for the purposes of analyses of commercial actors’ activities to influence policy-making processes (see, e.g. Miller *et al* 2023, O'Brien *et al*. 2023, Petticrew *et al*, 2018). Thirdly, our sample included a small number of industry submissions, and the submission website included four submissions which were confidential and unable to be accessed. It is not clear who these submissions were from and why they were confidential, but this may mean we may have not been able to see all industry submissions. Given that the pool of submissions for this study is small, further study is needed to assess how these mechanisms operate in the context of other alcohol policy inquiry and consultation processes and whether the framework might need to be modified for this sector. Future research could also examine any submissions made to the inquiry by organizations which are not themselves alcohol industry actors, but which receive funding from the alcohol industry, in order to explore whether the framing mechanisms and actions used in industry submissions are also reflected there.

The dissolution of the inquiry in light of the 2025 federal election stalled the progress of this inquiry and the generation of a comprehensive report with findings and recommendations. However, the issues paper from the inquiry, released in March 2025, recommended that ‘the successive Standing Committee on Health, Aged Care and Sport (or equivalent) in the 48th Parliament consider completing a full inquiry report into the health impacts of alcohol and other drugs in Australia’. The relevant Minister has now re-referred the inquiry to the Commonwealth House of Representatives Standing Committee on Health, Aged Care and Disability (as it now known) (new inquiry) which has the same terms of reference as the original inquiry. The new inquiry will consider all submissions made to the original inquiry. This means that the new inquiry will look at the alcohol industry submissions examined in this article. There are, as at 28th of October, only four new submissions to the new inquiry, none of which are from an alcohol industry actor. It will be important for future research to track the outcomes of the new inquiry to examine the influence of industry submissions on any findings and recommendations, and to follow the impact of the Committee’s report on policy in respect of alcohol.

## Conclusion

In conclusion, this analysis of alcohol industry submissions to the ‘Inquiry into the health impacts of alcohol and other drugs in Australia’ found the repetition of some common arguments and explored the framing mechanisms and actions through which alcohol industry actors create these frames. We found several of the framing mechanisms and actions identified by Campbell *et al*. (2020) in the context of food industry submissions reflected in alcohol industry submissions. We consider the framework to be a fruitful analytical tool for researchers and policy-makers alike. Finally, we also identified heightened and direct attacks on public health evidence in relation to alcohol with such attacks not seen within the Australian context previously.

## Supplementary Material

daaf223_Supplementary_Data

## Data Availability

All data are publicly available.
