# Supplementary material for: An analysis of framing mechanisms used in alcohol industry submissions to an Australian national parliamentary inquiry
Source: Health Promot Int. 2025 Dec 23;40(6):daaf223. doi: 10.1093/heapro/daaf223 (PMC12721998; doi:10.1093/heapro/daaf223)
Supplement: daaf223_Supplementary_Data [file daaf223_supplementary_data.docx]

| **Submission** | **Equating** | | **Contesting** | | **Dichotomising** | | **Cropping** | | |
| --- | --- | --- | --- | --- | --- | --- | --- | --- | --- |
|  | **Demanding Parity** | **Conflating** | **Co-opting** | **Exiting** | **Attributing** | **Boomeranging** | **Jockeying** | **Siloing** | **Blame-Shifting** |
| Alcohol Beverages Australia | ✓ | ✓ | ✓ | ✓ |  | ✓ |  | ✓ | ✓ |
| Brewers Association Australia | ✓ | ✓ | ✓ | ✓ |  |  |  | ✓ |  |
| Clubs Australia | ✓ | ✓ | ✓ | ✓ |  |  | ✓ |  |  |
| Independent Brewers Association | ✓ | ✓ | ✓ | ✓ |  |  | ✓ | ✓ | ✓ |
| Retail Drinks Australia | ✓ | ✓ | ✓ | ✓ |  | ✓ |  | ✓ | ✓ |
| Spirits & Cocktails Australia | ✓ | ✓ | ✓ |  |  | ✓ |  | ✓ | ✓ |
| ABAC Scheme Limited | ✓ | ✓ |  |  |  |  |  |  |  |
| DrinkWise | ✓ | ✓ | ✓ | ✓ |  | ✓ |  | ✓ |  |
| Harvest Advisory & Research |  |  |  | ✓ |  | ✓ |  |  |  |
| Australian Hotels Association [WA] |  | ✓ |  | ✓ |  | ✓ |  |  |  |

**Supplementary Table 1. Framing mechanisms and actions identified in each submission.**
